# Epidemiologic characteristics of oral cancer: single-center analysis of 4097 patients from the Sun Yat-sen University Cancer Center

**DOI:** 10.1186/s40880-016-0078-2

**Published:** 2016-03-03

**Authors:** Ji Zhang, Fan Gao, An-Kui Yang, Wen-Kuan Chen, Shu-Wei Chen, Huan Li, Xing Zhang, Zhong-Yuan Yang, Xin-Lin Chen, Ming Song

**Affiliations:** State Key Laboratory of Oncology in South China, Collaborative Innovation Center of Cancer Medicine, Sun Yat-sen University Cancer Center, 651 Dongfeng Road East, 510060 Guangzhou, Guangdong P. R. China; Department of Head and Neck Surgery, Sun Yat-sen University Cancer Center, 510060 Guangzhou, Guangdong P. R. China; Department of Intensive Care, Sun Yat-sen University Cancer Center, 510060 Guangzhou, Guangdong P. R. China; Department of Preventive Medicine and Biostatistics, School of Basic Medical Science, Guangzhou Higher Education Mega Center, Guangzhou University of Chinese Medicine, 232 Outer Ring East Road, 510006 Guangzhou, Panyu District, Guangdong P. R. China

**Keywords:** Oral cancer, Epidemiology, Guangdong

## Abstract

**Background:**

Oral cancer is a common type of head and neck cancers. Knowing its epidemiologic characteristics is crucial to preventing, diagnosing, and treating this cancer. This study aimed to explore the epidemiologic characteristics of oral cancer in South China.

**Methods:**

We retrospectively analyzed data from 4097 oral cancer patients treated at the Sun Yat-sen University Cancer Center between 1960 and 2013. We compared the age of onset, sex ratio, pathologic type, and primary tumor location among three subcultural areas (Guangfu, Hakka, and Chaoshan) and between an economically developed region and a less-developed one in Guangdong.

**Results:**

Overall, oral cancer had a male-to-female ratio of approximately 2:1, and this ratio decreased over time. Oral cancer occurred mostly in patients of 45–64 years old (54.5%), and the percentage of older patients gradually increased over time. The most common tumor location was the tongue. Squamous cell carcinoma was the predominant pathologic type. The percentage of blood type O in oral cancer patients was lower than that in the healthy population. The male-to-female ratio in the Chaoshan area was higher than that in the Guangfu and Hakka areas, whereas the age of disease onset in Guangfu was higher than that in Hakka and Chaoshan. The male-to-female ratio was lower and the age of disease onset was higher in the economically developed region than in the less-developed region.

**Conclusion:**

The incidence of oral cancer in South China presents typical characteristics to which doctors should pay attention when diagnosing and treating oral cancer patients.

## Background

More than two million deaths of cancer per year [1] make it a major public issue in China. Worldwide, the 263,900 cases of oral cancer per year make it the 10th most common cancer in men [2]. The incidence of oral cancer is widely believed to be associated with the use of tobacco, alcohol, betel quid (a vine with mild stimulant properties), and areca nut (a palm nut with mild stimulant properties often chewed with betel quid) [3]. People of lower socioeconomic class may also be more prone to oral cancer than those of higher class [4], perhaps because of poor oral hygiene and nutrition. Some researchers believe that the incidence is also associated with air pollution [5].

The Sun Yat-sen University Cancer Center is the largest cancer hospital in Guangdong Province and in South China and treats more cancer patients than most hospitals in this region. Therefore, patients treated in this center largely present with typical characteristics of diseases in this region, including oral cancer, which is a major disease diagnosed and treated here. We retrospectively analyzed data from patients with oral cancer treated at the cancer center to determine the disease characteristics prevalent in South China, with the aim of providing a sound knowledge base for its prevention, diagnosis, and treatment.

## Methods

### Patient collection

Data from all patients with oral cancer treated at the Sun Yat-sen University Cancer Center between 1960 and 2013 were collected. All the patients were diagnosed by pathologic analysis.

### Data collection

In addition to standard demographic data, we collected data on the date of hospital admission, location of the primary tumor, blood type, native place, long-term residence, and so on.

Patients were classified into five groups by period of admission (1960–1973, 1974–1983, 1984–1993, 1994–2003, and 2004–2013) and seven age groups (0–24, 25–34, 35–44, 45–54, 55–64, 65–74, and ≥75 years).

The location of the primary tumor in each patient was identified as being in one of six parts of the mouth: the body of the tongue (the front two-thirds of the tongue, not including the root), the floor of the mouth, the buccal mucosa, the alveolus, the retromolar area, and the hard palate (not including the soft palate).

Guangdong Province was divided into three subcultural, geographic areas according to residents’ dialects, diets, and living habits: Guangfu (also known as Cantonese, including Guangzhou, Shenzhen, Zhuhai, Foshan, Zhanjiang, Zhaoqing, Jiangmen, Maoming, Yangjiang, Qingyuan, Dongguan, Zhongshan, and Yunfu), Hakka (Meizhou, Huizhou, Heyuan, and Shaoguan), and Chaoshan (Chaozhou, Shantou, Jieyang, and Shanwei). Patients’ native places were classified into one of these three geographic areas.

Guangdong was further divided into two regions: the Pearl River Delta region (Guangzhou, Shenzhen, Zhuhai, Foshan, Zhaoqing, Jiangmen, Huizhou, Dongguan, and Zhongshan) and the non-Pearl River Delta region. The Pearl River Delta region is markedly more economically developed [6]. Patients’ long-term residences were classified in these two geographic regions.

### Statistical analysis

All data were sorted and analyzed with SPSS 21.0 (SPSS Inc., Chicago, IL, USA). Alpha was set at 0.05, and all tests were two-tailed. Frequencies and proportions were calculated for the demographic data. The Chi square test was used to analyze the associations of oral cancer with sex and blood type. The Analysis of Variance (ANOVA) was used when analyzing the age distribution. The Bonferroni correction was applied for multiple comparisons.

## Results

Data from 4097 patients, 2729 males and 1368 females, were analyzed (Table 1).

**Table 1:** Residential and hospital admission characteristics of 4097 oral cancer patients in South China by sex

Variate	Total	Male	Female	M:F ratio	*P*
Period of hospital admission	4097	2729	1368	1.99	<0.05
1960–1973	432	293	139	2.11	
1974–1983	500	344	156	2.21	
1984–1993	803	549	254	2.16	
1994–2003	959	635	324	1.96	
2004–2013	1403	908	495	1.83	
Subcultural area^a^	3630	2418	1212	2.00	<0.05
Guangfu (Cantonese)	2470	1602	868	1.85	
Hakka	488	315	173	1.82	
Chaoshan	481	340	141	2.41	
Unknown	191	161	30	5.37	
Economic region^b^	3661	2427	1234	1.97	<0.05
Pearl river delta region	2479	1617	862	1.88	
Non-pearl river delta region	1072	736	336	2.19	
Unknown	110	74	36	2.06	

^a^A total of 467 non-Cantonese patients were excluded from this analysis

^b^A total of 436 patients who live in other provinces were excluded from this analysis

### Age distribution

The median age of disease onset of the patients was 53 years (range, 3–97 years). The age of onset was concentrated between the ages of 45 and 64 years (Table 2). In addition, over the study period of approximately 50 years, the percentage of older patients significantly increased (*F* = 17.29, *P* < 0.001).

**Table 2:** Residential and hospital admission characteristics of 4097 oral cancer patients in South China by age at admission

Variate	Total	Age (years)							*P*
		0–24	25–34	35–44	45–54	55–64	65–74	75–	
Period of hospital admission	4097	96	287	711	1056	1178	622	147	<0.001
1960-1973	432	17	36	110	103	114	48	4	
1974-1983	500	10	44	83	171	131	54	7	
1984-1993	803	22	55	145	197	250	119	15	
1994-2003	959	16	68	157	236	270	162	50	
2004-2013	1403	31	84	216	349	413	239	71	
Subcultural area^a^	3630	86	239	623	927	1049	569	137	<0.001
Guangfu (Cantonese)	2470	46	152	402	612	740	421	97	
Hakka	488	16	33	95	142	129	58	15	
Chaoshan	481	12	42	95	134	133	50	15	
Unknown	191	12	12	31	39	47	40	10	
Economic region^b^	3661	86	251	630	929	1054	575	136	<0.001
Pearl river delta region	2479	41	145	394	627	738	433	101	
Non-pearl river delta region	1072	33	92	213	279	295	130	30	
Unknown	110	12	14	23	23	21	12	5	

^a^A total of 467 non-Cantonese patients were excluded from this analysis

^b^A total of 436 patients who live in other provinces were excluded from this analysis

### Tumor location

Most tumors were on the tongue (64.3%), followed by the gingiva, hard palate, buccal mucosa, floor of the mouth, lips, and retromolar area (Table 3).

**Table 3:** Disease characteristics of 4097 oral cancer patients in South China

Variate	Number of cases	Percentage (%)
Tumor location	4097	
Body of tongue	2230	54.4
Floor of mouth	305	7.4
Buccal mucosa	367	9.0
Gingiva	534	13.0
Retromolar area	18	0.4
Hard palate	381	9.3
Lips	187	4.6
Unknown	75	1.8
Pathologic type	4097	
Squamous cell carcinoma	3642	88.9
Adenoid cystic carcinoma	119	2.9
Mucoepidermoid carcinoma	77	1.9
Malignant melanoma	48	1.2
Adenocarcinoma	32	0.8
Basaloid carcinoma	28	0.7
Malignant mixed tumor	25	0.6
Sarcoma	22	0.5
Myoepithelial carcinoma	4	0.1
Others	100	2.4
Blood type	4097	
A	1021	24.9
B	1039	25.4
AB	253	6.2
O	1519	37.1
Unknown	265	6.5

### Pathologic type

Squamous cell carcinoma was the most common type of oral cancer (88.9%; Table 3). Of the 3642 cases of squamous cell carcinoma, 2126 were highly differentiated, 928 were moderately differentiated, 289 were poorly differentiated, and 299 were unknown.

### Blood type

The blood type of 265 patients was not identified. The distribution of blood types of the remaining patients is presented in Table 3.

### Distribution of native places

Most patients were Cantonese (3630 patients). The distributions of Cantonese patients by their native places were Guangfu (2470), Hakka (488), Chaoshan (481), and unknown (191).

The male-to-female ratio differed significantly in the three subcultural areas (*χ*^*2*^ = 6.36, *P* < 0.05). Pairwise comparisons indicated that the ratio in the Chaoshan area was significantly higher than those in the Hakka and Guangfu areas (Table 1).

The age of disease onset also differed significantly among the three subcultural areas (*F* = 9.96, *P* < 0.001). Pairwise comparisons indicated that the age of disease onset in Guangfu was significantly higher than those in Hakka and Chaoshan (Table 2).

### Economic conditions

A total of 436 patients who live in other provinces were excluded from this analysis. Of the 3661 residents in Guangdong, 2479 lived in the Pearl River Delta region, 1072 lived in the non-Pearl River Delta region, and 110 were unknown. The male-to-female ratio differed significantly between these two regions (*χ*^*2*^ = 3.94, *P* < 0.05), with a higher ratio in the Pearl River Delta region (Table 1). Age at disease onset differed significantly between the Pearl River Delta and non-Pearl River Delta regions (*F* = 31.51, *P* < 0.001), with an older age at disease onset in the Pearl River Delta region (Table 2).

## Discussion

According to our study, the incidence of oral cancer differs by sex, age, blood type, life style, and economic condition.

The incidence of oral cancer in males was significantly higher than that in females. The male-to-female ratio is 10.5 in Taiwan, China [7] and 1.42 in the United States [8]. The male-to-female ratio in the present study was approximately two. This disparity may result from sex differences in exposure to risk factors for oral cancer [8, 9]. For example, men generally consume more alcohol and cigarettes than women.

In the present study, the male-to-female ratio showed a slightly downward trend, and the percentage of female oral cancer patients gradually increased from 32.2% in 1960s to 35.3% now. Other investigators have reported similar findings. For example, the worldwide epidemiologic study of oral cancer by Warnakulasuriya et al. [9] found that the male-to-female ratio of oral cancer had decreased in recent decades, which might be associated with changes in the degree of exposure to risk factors.

In our study, half the patients were between 45 and 64 years old. This result is consistent with most other reports. For example, Wen et al. [10] reported that the highest prevalence was between the ages of 41 and 60 years.

In the present study, over approximately 50 years, the percentage of younger patients with oral cancer gradually decreased, whereas the percentage of older patients gradually increased. This change may be explained by the fact that, in recent decades, the Chinese economy has been rapidly expanded and living conditions have been rapidly improved. Additionally, due to the implementation of family planning policies, the size of the aging population has increased, with a consequent increase in the proportion of older people in the population. Increased life span, resulting from the improved living conditions, has further increased this proportion. Therefore, the increase in the proportion of older patients may not reflect an actual increase in the incidence in older people.

The most common tumor sites of oral cancer in the United States are the tongue, alveolus, and lips [11]. Data from Southeast China showed that the three most common locations were the tongue, floor of the mouth, and gingiva in males and the tongue, buccal mucosa, and gingiva in females [12]. In the present study, we found that two-thirds of tumors were on the tongue, followed by the gingiva, hard palate, buccal mucosa, floor of the mouth, lips, and retromolar area.

As in most other reports, our study also found that the cancer type with the highest incidence was squamous cell carcinoma, which accounted for nearly 90% of cancers. In West China, approximately two-thirds of oral and maxillofacial malignant tumors were squamous cell carcinomas [10]. Other common types in our study were malignant lymphoma, mucoepidermoid carcinoma, adenoid cystic carcinoma, and adenoma. However, we found fewer lymphomas than that reported in West China, perhaps because we excluded cases of cancer in the oropharynx, which includes locations such as the tonsils, soft palate, and root of the tongue.

Many reports have shown that the ABO blood type system is associated with the development of cancers. Type A has been associated with gastric [13–15], pancreatic [16–19], ovarian [16, 20], esophageal [21], salivary gland [21], and breast cancers [16], whereas type B has been associated with esophageal [22, 23] and laryngeal cancers [21]. Some reports showed that people with blood type O have lower risks for lung [24], endometrial [25], pancreatic [26], renal cell [27], and ovarian cancers [28], and colorectal adenocarcinoma [29]. However, we found no studies that assessed the association between blood type and oral cancer. We combined the data for types A, B, and AB (type other) and compared the proportions of blood types for our patients with control data from a representative sample of the Guangdong population published by Chen et al. [30]. Of our patients, 1519 had type O and 2313 had type other, whereas in the control group, 10,702 had type O and 14,210 had type other. The proportion of oral cancer patients with type O blood was significantly lower than that of controls (*χ*^*2*^ = 14.97, *P* < 0.001).

In this study, almost 90% of our patients were Cantonese. There are three major subcultural areas in Guangdong province: Guangfu, Hakka, and Chaoshan. The dialects, diets, and living habits in these three areas differ markedly. The male-to-female ratio of oral cancer patients in the Chaoshan area was significantly higher than that in the Guangfu and Hakka areas, the possible reasons for which are as follows. Smoking and drinking are more prevalent in the Chaoshan area, with men more often addicted to drinking and smoking than women. It is well known that drinking [31, 32] and smoking [31, 33–35] promote the development and progression of oral cancer. In addition, in most families in the Chaoshan area, men are absolutely dominant; therefore, if women experience symptoms, they might remain silent, or their complaint may not receive enough attention from family members, leading to lower diagnosis and treatment rates in large hospitals.

The age of onset in patients from the Guangfu area was older than that of the other two areas. It is possible that the lifestyle habits of people in Hakka and Chaoshan more likely lead to early exposure to carcinogenic factors. For example, Chaoshan people like to drink very hot Kungfu tea (often approximately 75 °C or 167 °F). Hakka cuisine also stresses that food should be eaten when it is hot. Eating very hot food or tea has been reported to increase the risk of gastrointestinal tumors through mucosal damage, inflammatory factors, and heat shock protein activity [36, 37]. The high incidence of esophageal cancer and laryngeal cancer in this area might be associated with this habit. Hakka cuisine uses more salt and oil. Excessive salt or oil intake is associated with the development of tumors [38]. Chaoshan people like pickled food, fish sauce, and barbecued food, which are considered to have cancer-promoting effects [38, 39]. The prevalence of smoking and drinking in the Chaoshan area could also significantly increase the incidence of oral cancer.

The Pearl River Delta region is markedly more economically developed than the rest of Guangdong province. The male-to-female ratio of oral cancer patients in the non-Pearl River Delta region was significantly higher than that in the Pearl River Delta region, possibly because bad health habits, such as drinking and smoking, were more common in people, mainly males, in the economically underdeveloped regions.

The mean age of disease onset in the Pearl River Delta region was higher than that in the non-Pearl River Delta region. It is possible that people in the economically underdeveloped regions had a higher chance of encountering carcinogenic factors at an early age than those in the developed regions. For example, a poor working environment [40], poor nutritional status and developmental retardation [36, 41], a low education level [38], low awareness of dental care and poor oral hygiene [42], common poor health habits (such as excessive drinking and smoking), few physical examinations for cancer prevention, and low levels of medical care [40] would affect the age distribution of oral cancer patients.

This study design is a retrospective single center analysis, so an unavoidable problem is missing data. We drew conclusions about the epidemiologic features of oral cancer in different areas from some indirect evidence. Prospective controlled studies need be carried out to support our conclusions.

## Conclusions

We infer from our data that during the process of screening for oral cancer, clinicians should pay attention to middle-aged and older people, men, and individuals with non-O blood type. Physicians should pay attention to every corner of the oral cavity and focus on examining sites of predilection. To help prevent the development of oral cancer, targeted information should be disseminated to encourage residents to change poor lifestyle habits. We should also appeal to the government to increase investment in medical care in economically underdeveloped regions.
